# Prognostic impact of CT-derived muscle-adipose index change in patients with inoperable esophageal squamous cell carcinoma undergoing chemoradiotherapy

**DOI:** 10.3389/fonc.2025.1652384

**Published:** 2025-10-29

**Authors:** Bingyi Zhang, Dong Guo, Yuanyuan Cai, Xiaoxiao Wang, Xinyue Wei, Yang Li, Xiaoli Wang, Jianwen Li, Furong Hao

**Affiliations:** Department of Radiation Oncology, Weifang People’s Hospital, Shandong Second Medical University, Weifang, Shandong, China

**Keywords:** muscle-adipose index changes, esophageal squamous cell carcinoma, computed tomography, chemoradiotherapy, prognostic biomarker

## Abstract

**Background:**

The muscle-adipose index (MAI), a novel nutritional parameter for assessing body composition, has emerged as a potential prognostic indicator. This study aimed to research MAI and its longitudinal changes before and after chemoradiotherapy (CRT) and to evaluate the prognostic implications of these changes in patients with inoperable esophageal squamous cell carcinoma (ESCC).

**Methods:**

This retrospective cohort included 180 ESCC patients treated with CRT (2020-2024). MAI was derived from CT-based quantification of skeletal muscle and subcutaneous adipose tissue at the third lumbar vertebra(L3). Baseline (preMAI), post-treatment (postMAI), and their longitudinal changes (ΔMAI) were analyzed. Optimal cutoff values for MAI imbalance were determined using X-tile software. Overall survival (OS) and progression-free survival (PFS) were assessed using Kaplan-Meier and Cox regression analyses.

**Results:**

Among 180 enrolled patients, 111 (61.7%) patients died during follow-up (median OS:23.0 months; median PFS:16.0 months).PreMAI and postMAI demonstrated statistically significant associations with OS (preMAI: HR = 5.934,95%CI=3.943-8.929, P<0.001; postMAI: HR = 9.123,95%CI=5.769-14.426, P<0.001) and PFS (preMAI: HR = 5.316, 95%CI=3.583-7.889, P<0.001; postMAI: HR = 8.008, 95%CI=5.213-12.303, P<0.001). The 0 group (_pre_balance-_post_balance) demonstrated significantly better survival outcomes than the remaining groups, both in terms of OS (HR = 9.454, 95%CI=5.830-15.331, P<0.001) and PFS (HR = 8.444, 95%CI=5.360-13.303, P< 0.001). Multivariate analysis confirmed ΔMAI as an independent prognostic factor for OS (HR = 2.953, 95%CI=1.070-8.151, P = 0.037) and PFS (HR = 3.204, 95%CI=1.166-8.806, P = 0.024).

**Conclusion:**

CT-derived MAI was a robust prognostic biomarker in ESCC. These findings highlighted the clinical utility of MAI for risk stratification and personalized therapeutic strategies in inoperable ESCC patients.

## Introduction

Esophageal cancer ranks as the seventh leading cause of cancer mortality globally (1). In China, esophageal squamous cell carcinoma (ESCC) accounts for approximately 90% of esophageal cancer cases (2). A significant proportion of ESCC patients are considered ineligible for surgery due to advanced-stage disease at diagnosis, comorbidities, or patient-specific factors (3, 4). For patients with inoperable locally advanced ESCC, definitive chemoradiotherapy (CRT) remains the primary treatment; however, the 5-year overall survival (OS) rate remains suboptimal (approximately 20-40%) (5, 6). ESCC patients are prone to nutritional impairment due to dysphagia, cancer cachexia, treatment-related toxicities, and psychological comorbidities (7). Therefore, comprehensive nutritional assessment enables risk stratification, improves prognosis prediction, and guides personalized clinical decisions (8).

Traditional nutritional indices, including body mass index (BMI), serum albumin (ALB), patient-generated subjective global assessment (PG-SGA), and CONUT score, served as cornerstone metrics for comprehensive nutritional status evaluation (9). However, these metrics exhibit limitations in comprehensiveness, as they focus on specific aspects of nutrition. Thus, researchers have increasingly recognized the value of body composition analysis in nutritional assessment. Recent advancements in image analysis algorithms allow precise quantification of skeletal muscle mass and subcutaneous adipose tissue through CT scans, providing innovative biomarkers for nutritional assessment. Higher skeletal muscle mass is associated with better treatment tolerance, reduced complication rates, and improved survival in oncology patients (10). Sarcopenia was associated with adverse outcomes across various malignancies, including higher postoperative complication rates and poorer survival in esophageal cancer, as well as increased treatment toxicity and reduced survival in head and neck cancers (11, 12). However, subcutaneous adipose tissue demonstrates dual prognostic effects across cancers. Higher subcutaneous adipose area associates with improved OS and progression-free survival (PFS) in colorectal cancer (13), whereas it correlates with worse survival in ovarian cancer (14). However, most studies have employed isolated muscle or adipose indices, with limited reports on the combined prognostic value of muscular and adipose measurements in oncology. Recent studies demonstrated that preoperative muscle attenuation index (MAI) correlates with survival outcomes in gastric cancer patients (15). Notably, the CT-derived Muscle-Adipose Index Change (ΔMAI) may better reflect dynamic nutritional alterations, yet its prognostic significance in ESCC remains unexplored.

This study aimed to evaluate the prognostic significance of preMAI, postMAI and ΔMAI in patients with inoperable ESCC. Additionally, we compared the predictive performance of ΔMAI with conventional nutritional indices to determine their predictive value on survival outcomes.

## Materials and methods

### Study cohort

**Figure 1:**
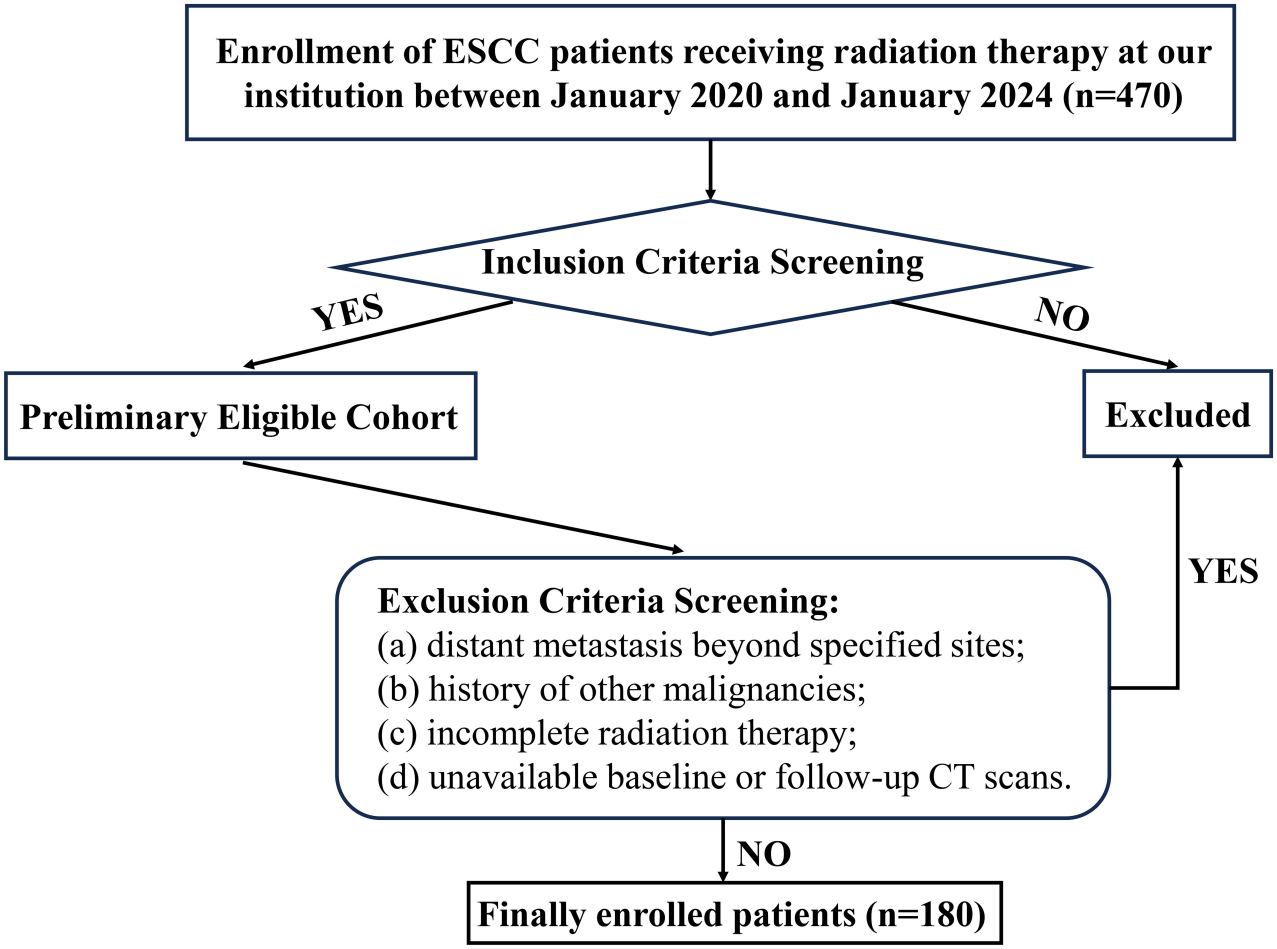
Patient screening and enrollment flowchart.

This review included 180 consecutive ESCC patients treated with CRT at Weifang People’s Hospital between January 2020 and January 2024. Inclusion criteria comprised: (a)histopathologic confirmation of ESCC via endoscopic biopsy; (b) Patients with stage II or III disease who decline surgery; T4b disease; thoracic ESCC with supraclavicular or retroperitoneal lymph node metastasis only; or involvement of non-regional mediastinal lymph nodes; (c)completed the full course of radiation therapy; (d)Karnofsky Performance Status (KPS)≥70; (e)complete clinical records and follow-up data. Exclusion criteria: (a)distant metastasis beyond specified sites; (b)history of other malignancies; (c)incomplete radiation therapy; (d)unavailable baseline or follow-up CT scans. A detailed enrollment flowchart is provided in **Figure 1**. Ethical approval was granted by the Institutional Review Board of Weifang People’s Hospital (Approval No. KYLL20250530-4). The requirement for informed consent was waived because the data were anonymized.

### Treatment protocols

Radiation therapy was delivered using intensity-modulated radiotherapy (IMRT). Target volumes were delineated according to the Chinese Esophageal Cancer Radiotherapy Target Delineation Guidelines and the NCCN Guidelines for Esophageal and Esophagogastric Junction Cancers. The total prescription dose ranged from 50 to 60 Gy, delivered in 25–30 fractions of 2 Gy per fraction. The standard concurrent chemotherapy regimen consisted of paclitaxel and carboplatin administered weekly. Patients aged≥70 years or with KPS≥70 received monotherapy with oral tegafur or capecitabine, based on physician’s assessment of tolerance.

### Data collection

Clinical and pathological parameters were extracted from patient medical records. Baseline characteristics included age, sex, smoking history, alcohol consumption history, KPS, serum albumin, total cholesterol, carbohydrate antigen19-9(CA19-9), carcinoembryonic antigen (CEA), and absolute counts of neutrophils, lymphocytes, and monocytes. Baseline tumor characteristics-including site, T stage, N stage, and pathological features-were assessed via esophagoscopy and contrast-enhanced CT of the thorax and abdomen prior to treatment initiation.

### Muscle-adipose index quantification

**Figure 2:**
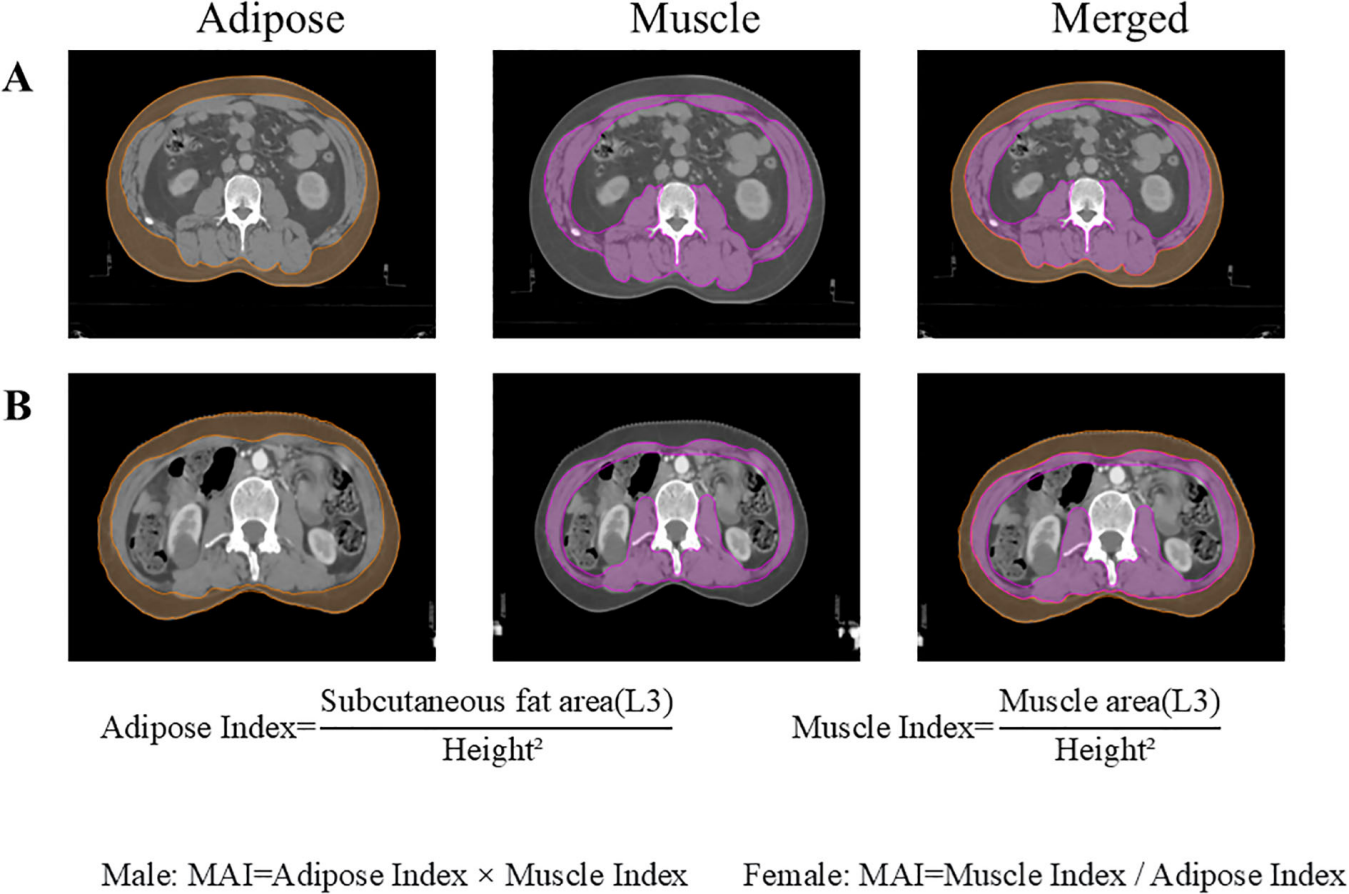
Cross-sectional areas of skeletal muscle and subcutaneous adipose tissue at the L3 vertebral level. **(A)** A 48-year-old male patient with ESCC; **(B)** A 61-year-old female patient with ESCC. ESCC, esophageal squamous cell carcinoma.

Axial CT images at the third lumbar vertebra (L3) were analyzed to measure cross-sectional areas (cm²) of skeletal musculature (encompassing the abdominal wall and paraspinal muscles) and subcutaneous adipose tissue at the umbilical level (16). Using 3D Slicer software (v5.8.1) calculated muscle and fat indices normalized to height² (cm²/m²). Given the distinct prognostic significance of adipose indices between male and female patients, MAI was defined as the product of adipose index and muscle index in males, while calculated as the ratio of muscle index to adipose index in females (15). As shown in **Figure 2**, MAI (male)= Adipose Index × Muscle Index; MAI (female)=Muscle Index/Adipose Index. Diagnostic cutoffs for muscle-adipose imbalance were established using X-tile software (v3.6.1), with the optimal cutoff selected to maximize the difference in 5-year overall survival between the groups. Patients with values above the diagnostic threshold were classified as balanced. Those below the threshold were classified as imbalanced.

### The definition of ΔMAI

Using the defined cut-off point, we categorized patients as having balanced or imbalanced MAI. These patients were further stratified into three groups based on ΔMAI, defined throughout the text as:0 group (_pre_balance-_post_balance), 1 group (_pre_balance-_post_imbalance or _pre_imbalance-_post_balance) and 2 group (_pre_imbalance- _post_imbalance).

### Follow-up protocol

Evaluations included tumor marker assessments, upper gastrointestinal contrast studies, contrast-enhanced CT (neck/chest/abdomen), and endoscopy at 3-to 6-month intervals for the first 2 years post-CRT, every 6 months during years 3-5, and annually thereafter. Survival status and recurrence data were collected through telephone interviews. Patient confidentiality was strictly maintained throughout all procedures. OS was determined from the treatment commencement date until death or the last recorded follow-up, whichever occurred first. PFS was calculated from the treatment start date to the date of disease progression, death, or the final follow-up, whichever occurred first. Follow-up extended until death or December 31, 2024.

### Statistical analysis

All statistical analyses were performed using GraphPad Prism (version 10.4.1). Continuous variables are reported as mean ± standard deviation (SD) or median (interquartile range; range). Categorical variables are presented as frequencies (%). Categorical variables were compared using the chi-square test. Kaplan-Meier estimates and log-rank tests evaluated OS and PFS. Cox proportional hazards models identified prognostic factors associated with survival outcomes. P<0.05 was considered statistically significant.

## Results

### Patient and tumor characteristics

Baseline characteristics of all patients are summarized in **Table 1**. The study included 180 patients diagnosed with inoperable ESCC (mean age at diagnosis: 66.3 years; 137(76.1%) male, 43(23.9%) female). The mean pretreatment adipose and muscle indices were 38.69 cm²/m² and 41.77 cm²/m², respectively, which decreased to 39.98 cm²/m² and 33.93 cm²/m² posttreatment. Among the cohort, 36 patients (20.0%) were classified as stage II, 103 (57.2%) as stage III, and 41 (22.8%) as stage IV.

**Table 1:** Baseline characteristics of all patients.

Variables	N (%)
Age (years)	
<65	69 (38.3)
≥65	111 (61.7)
Gender	
Female	43 (23.9)
Male	137 (76.1)
Smoking history	
Never	74 (41.1)
Current or former	106 (58.9)
Alcohol consumption history	
Never	86 (47.8)
Current or former	94 (52.2)
KPS	
90-100	132 (73.3)
70-90	48 (26.7)
Tumor location	
Upper/Middle	116 (64.4)
Lower	64 (35.6)
TNM stage	
vII/III	139 (77.2)
IV	41 (22.8)
T stage	
T1-2	21 (11.7)
T3-4	159 (88.3)
N stage	
N0-1	106 (58.9)
N2-3	74 (41.1)
Differentiation grade	
Well/Moderate	47 (26.1)
Poor	133 (73.9)
NLR	
<8.83	114 (63.3)
≥8.83	66 (36.7)
SII	
<853.39	108 (60.0)
≥853.39	72 (40.0)
PNI	
<49.63	93 (51.7)
≥49.63	87 (48.3)
CEA	
<5ng/ml	130 (72.2)
≥5ng/ml	50 (27.8)
CA199	
<34U/ml	134 (74.4)
≥34U/ml	46 (25.6)
preMAI	
Balance	68 (37.8)
Imbalance	112 (62.2)
postMAI	
Balance	51 (28.3)
Imbalance	129 (71.7)
ΔMAI	
0 group	12 (6.7)
1 group	97 (53.9)
2 group	71 (39.4)

KPS, Karnofsky Performance Status; NLR, neutrophil-to-lymphocyte ratio; SII, systemic immune-inflammation index, PNI, prognostic nutritional index; CEA, carcinoembryonic antigen; CA199, carbohydrate antigen19-9; preMAI, Pre-therapy Muscle-Adipose Index; postMAI, Post-therapy Muscle-Adipose Index; ΔMAI, Change in Muscle-Adipose Index.

### Association of preMAI, postMAI, and ΔMAI with clinical features

The optimal cut-off values for MAI, determined using X-tile software based on survival outcomes, were 1239.9 for males and 0.5 for females in the pre-treatment assessment. In the post-treatment assessment, the cut-offs were 1269.7 for males and 0.5 for females. Based on pre- and post-treatment MAI values, enrolled patients were classified into MAI balanced and MAI imbalanced groups. The preMAI groups included 108 (60.0%) balanced and 72 (40.0%) imbalanced cases, while the postMAI distribution showed 99 (55.0%) balanced and 81 (45.0%) imbalanced cases. The relationships between preMAI/postMAI and clinicopathologic features are summarized in **Table 2**. Results revealed significant differences in differentiation grade between preMAI balanced and preMAI imbalanced groups (all P<0.05). PostMAI imbalance showed significant associations with elevated neutrophil-to-lymphocyte ratio (NLR), systemic immune-inflammation index (SII), advanced TNM stage, and poorer tumor differentiation grade (all P<0.05). PreMAI and postMAI values showed no significant associations with age, sex, smoking history, alcohol consumption history, KPS, tumor location, prognostic nutritional index (PNI), CA19–9 and CEA (all P>0.05).

**Table 2:** Correlation between MAI and clinicopathological parameters.

Variables	preMAI		P value	postMAI		P value
	Balance(68)	Imbalance(112)		Balance(51)	Imbalance(129)	
Age (years)			0.055			0.241
<65	20 (29.4%)	49 (43.75%)		23 (45.1%)	46 (35.7%)	
≥65	48 (70.6%)	63 (56.25%)		28 (54.9%)	83 (64.3%)	
Gender			0.527			0.751
Female	18 (26.5%)	25 (22.3%)		13 (25.5%)	30 (23.3%)	
Male	50 (73.5%)	87 (77.7%)		38 (74.5%)	99 (76.7%)	
Smoking history			0.765			0.494
Never	27 (39.7%)	47 (42.0%)		23 (45.1%)	51 (39.5%)	
Current or former	41 (60.3%)	65 (58.0%)		28 (54.9%)	78 (60.5%)	
Alcohol consumption history			0.875			0.589
Never	33 (48.5%)	53 (47.3%)		26 (51.0%)	60 (46.5%)	
Current or former	35 (51.5%)	59 (52.7%)		25 (49.0%)	69 (53.5%)	
KPS			0.963			0.331
90-100	50 (73.5%)	82 (73.2%)		40 (78.4%)	92 (71.3%)	
70-90	18 (26.5%)	30 (26.8%)		11 (21.6%)	37 (28.7%)	
Tumor location			0.954			0.461
Upper/Middle	24 (35.3%)	40 (35.7%)		16 (31.4%)	48 (37.2%)	
Lower	44 (37.9%)	72 (64.3%)		35 (68.6%)	81 (62.8%)	
TNM stage			0.201			0.302
II/III	56 (82.4%)	83 (74.1%)		42 (84.2%)	97 (75.2%)	
IV	12 (17.6%)	29 (25.9%)		9 (17.6%)	32 (24.8%)	
T stage			0.975			0.037
T1-2	8 (11.8%)	13 (11.6%)		10 (19.6%)	11 (8.5%)	
T3-4	60 (88.20%)	99 (88.4%)		41 (80.4%)	118 (91.5%)	
N stage			0.216			0.745
N0-1	44 (64.7%)	62 (55.4%)		31 (60.8%)	75 (58.1%)	
N2-3	24 (35.3%)	50 (44.6%)		20 (39.2%)	54 (41.9%)	
Differentiation grade			0.004			0.526
Well/Moderate	26 (38.2%)	21 (18.75%)		15 (29.4%)	32 (24.8%)	
Poor	42 (61.8%)	91 (81.25%)		36 (70.6%)	97 (75.2%)	
NLR			0.349			0.257
<8.83	46 (67.6%)	68 (60.7%)		29 (56.9%)	85 (65.9%)	
≥8.83	22 (32.4%)	44 (39.3%)		22 (43.1%)	44 (34.1%)	
SII			0.490			0.418
<853.39	43 (63.2%)	65 (58.0%)		33 (64.7%)	75 (58.1%)	
≥853.39	25 (36.8%)	47 (42.0%)		18 (35.3%)	54 (41.9%)	
PNI			0.134			0.150
<49.63	40 (58.8%)	53 (47.3%)		22 (43.1%)	71 (55.0%)	
≥49.63	28 (41.2%)	59 (52.7%)		29 (56.9%)	58 (45.0%)	
CEA			0.469			0.951
<5ng/ml	47 (69.1%)	83 (74.1%)		37 (72.5%)	93 (72.1%)	
≥5ng/ml	21 (30.9%)	29 (25.9%)		14 (27.5%)	36 (27.9%)	
CA199			0.826			0.990
<34U/ml	50 (73.5%)	84 (75.0%)		38 (74.5%)	96 (74.4%)	
≥34U/ml	18 (26.5%)	28 (25.0%)		13 (25.5%)	34 (25.6%)	

KPS, Karnofsky Performance Status; NLR, neutrophil-to-lymphocyte ratio; SII, systemic immune-inflammation index, PNI, prognostic nutritional index; CEA, carcinoembryonic antigen; CA199, carbohydrate antigen19-9; preMAI, Pre-therapy Muscle-Adipose Index; postMAI, Post-therapy Muscle-Adipose Index.

Among patients with balanced preMAI, 16 (14.8%) transitioned to MAI imbalance after treatment, while 92 (85.2%) maintained stable MAI status. Based on ΔMAI before and after treatment, all patients were stratified into 0 group (92,51.1%), 1 group (23,12.8%) and 2 group (65,36.1%) (**Table 3**). The results revealed that ΔMAI associated with tumor differentiation grade (P = 0.003). The factors including age, gender, smoking history, alcohol consumption history, KPS, tumor location, TNM stage, T stage, N stage, NLR, SII, PNI, CEA, and CA199 showed no significant correlation with ΔMAI (all P>0.05).

**Table 3:** Association between ΔMAI and clinicopathological characteristics.

Variables	ΔMAI			P value
	0 group (12)	1 group (97)	2 group (71)	
Age (years)				0.513
<65	3 (25.0%)	40 (41.2%)	26 (36.6%)	
≥65	9 (75.0%)	57 (58.8%)	45 (63.4%)	
Gender				0.781
Female	3 (25.0%)	25 (25.8%)	15 (21.1%)	
Male	9 (75.0%)	72 (74.2%)	56 (78.9%)	
Smoking history				0.934
Never	5 (41.7%)	41 (42.3%)	28 (39.4%)	
Current or former	7 (58.3%)	56 (57.7%)	43 (60.6%)	
Alcohol consumption history				0.841
Never	6 (50.0%)	48 (49.5%)	32 (45.1%)	
Current or former	6 (50.0%)	49 (50.5%)	39 (54.9%)	
KPS				0.933
90-100	9 (75.0%)	72 (74.2%)	51 (71.8%)	
70-90	3 (25.0%)	25 (25.8%)	20 (28.2%)	
Tumor location				0.487
Upper/Middle	4 (33.3%)	31 (32.0%)	29 (40.8%)	
Lower	8 (66.7%)	66 (68.0%)	42 (59.2%)	
TNM stage				0.035
II/III	9 (75.0%)	82 (84.5%)	48 (67.6%)	
IV	3 (25.0%)	15 (15.5%)	23 (32.4%)	
T stage				0.243
T1-2	3 (25.0%)	12 (12.4%)	6 (8.5%)	
T3-4	9 (75.0%)	85 (87.6%)	65 (91.5%)	
N stage				0.307
N0-1	7 (58.3%)	62 (63.9%)	37 (52.1%)	
N2-3	5 (41.7%)	35 (36.1%)	34 (47.9%)	
Differentiation grade				0.012
Well/Moderate	4 (33.3%)	33 (34.0%)	10 (14.1%)	
Poor	8 (66.7%)	64 (66.0%)	61 (85.9%)	
NLR				0.044
<8.83	4 (33.3%)	67 (69.1%)	43 (60.6%)	
≥8.83	8 (66.7%)	30 (30.9%)	28 (39.4%)	
SII				0.498
<853.39	7 (58.3%)	62 (63.9%)	39 (54.9%)	
≥853.39	5 (41.7%)	35 (36.1%)	32 (45.1%)	
PNI				0.612
<49.63	7 (58.3%)	44 (45.4%)	36 (50.7%)	
≥49.63	5 (41.7%)	53 (54.6%)	35 (49.3%)	
CEA				0.539
<5ng/ml	7 (58.3%)	71 (73.2%)	52 (73.2%)	
≥5ng/ml	5 (41.7%)	26 (26.8%)	19 (26.8%)	
CA199				0.404
<34U/ml	7 (58.3%)	74 (76.3%)	53 (74.6%)	
≥34U/ml	5 (41.7%)	23 (23.7%)	18 (25.4%)	

HR, Hazard ratio; CI, Confidence interval; OS, overall survival; PFS, progression-free survival; KPS, Karnofsky Performance Status; TNM stage, Tumor Node Metastasis; NLR, neutrophil-to-lymphocyte ratio; SII, systemic immune-inflammation index, PNI, prognostic nutritional index; CEA, carcinoembryonic antigen; CA199, carbohydrate antigen19-9; ΔMAI, Change in Muscle-Adipose Index.

### Impact of preMAI, postMAI, and ΔMAI on prognosis

**Figure 3:**
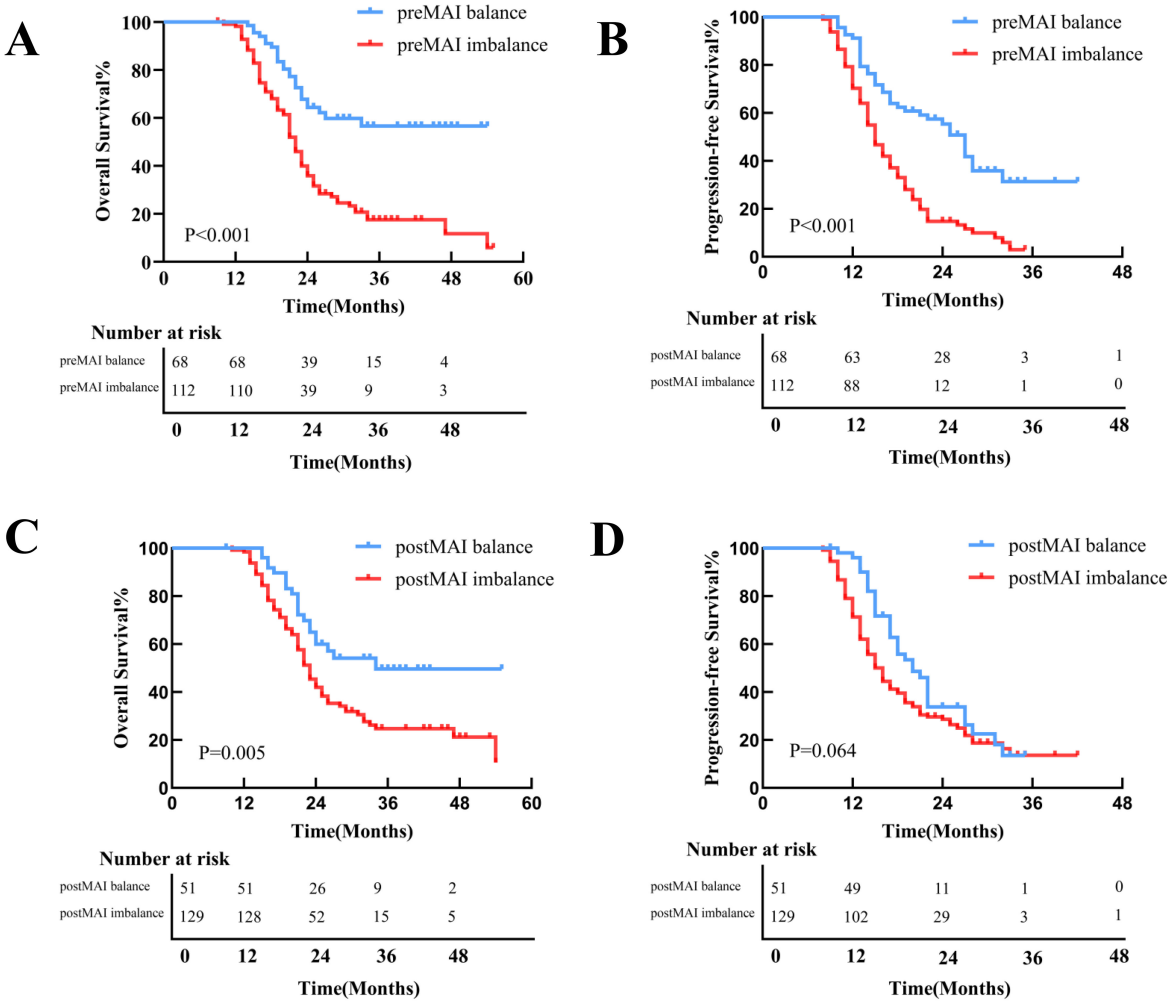
Kaplan-Meier Curves for OS and PFS Stratified by preMAI and postMAI Status. **(A)** The preMAI balanced group demonstrated significantly OS compared to the preMAI imbalanced group (P<0.001). **(B)** The preMAI balanced group showed significantly superior PFS versus the preMAI imbalanced group (P<0.001). **(C)** The postMAI balanced group exhibited significantly longer OS than the postMAI imbalanced group (P<0.001). **(D)** The postMAI balanced group had significantly improved PFS relative to the postMAI imbalanced group (P<0.001). MAI, muscle-adipose index. OS, overall survival. PFS, progression-free survival.

**Figure 4:**
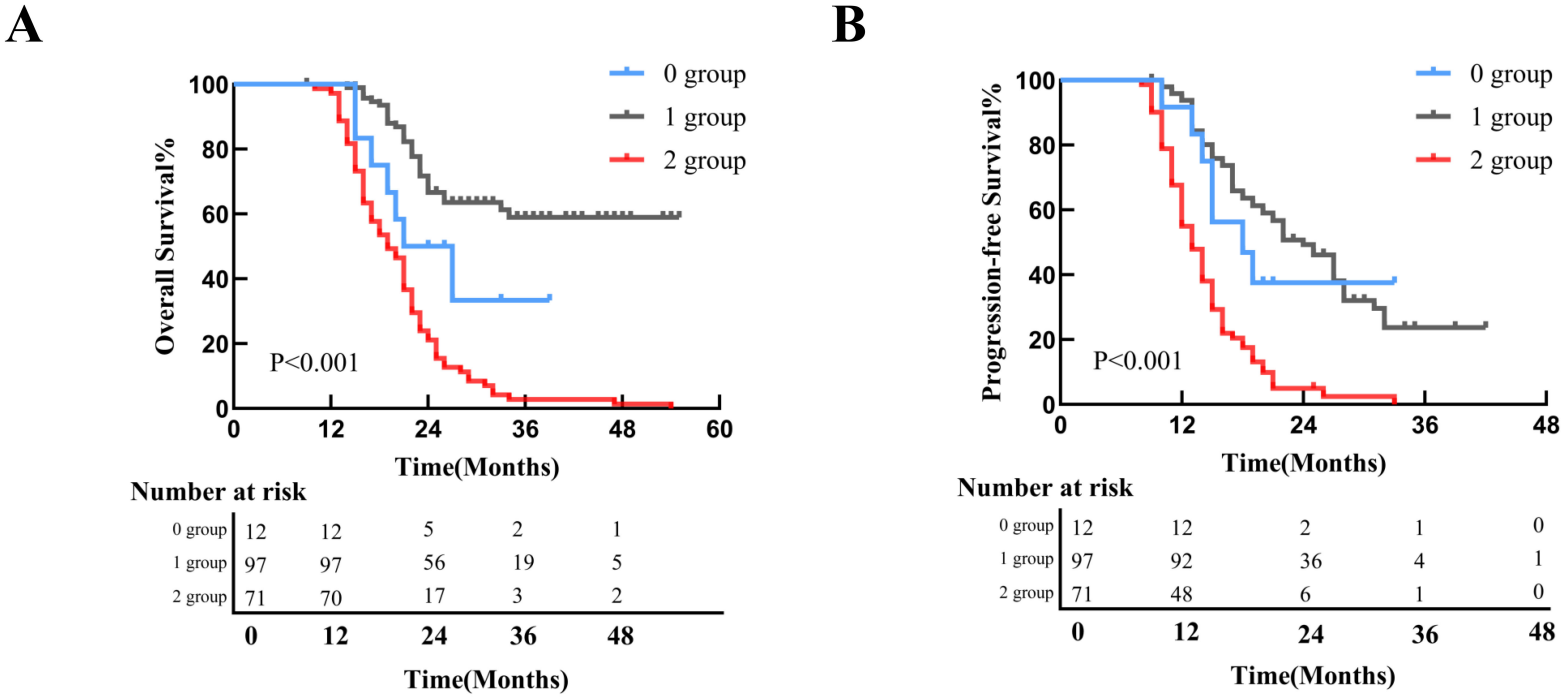
Survival Outcomes Stratified by ΔMAI Groups. **(A)** Kaplan-Meier curve for overall survival (OS) in all patients; **(B)** Kaplan-Meier curve for progression-free survival (PFS) in all patients. MAI, muscle-adipose index.

With a median follow-up of 31 months (range: 3–55 months), 111 patients (61.7%) died, and 36 (20.0%) experienced disease progression without death. Median OS and PFS for the entire cohort were 23.0 months and 16.0 months. Kaplan-Meier survival curves demonstrated superior OS and PFS in preMAI balanced groups than imbalanced groups (**Figures 3A, B**). Median OS was 25 months in the preMAI balanced group and 16.5 months in the imbalanced group (P<0.001). Similarly, postMAI balanced patients exhibited significantly better OS and PFS than imbalanced groups (**Figures 3C, D**). Survival analysis of ΔMAI groups revealed significantly inferior OS and PFS in patients with 1 group and 2 group compared to the 0 group (OS: HR = 9.454; 95%CI=5.830-15.331; P<0.001; PFS: HR = 8.444; 95%CI=5.360-13.303; P<0.001) (**Figure 4** **).**

### Prognostic factor analysis

Univariate Cox regression analysis identified multiple factors associated with OS and PFS (**Tables 4** and **5**). Advanced TNM stage (P = 0.002), poor differentiation grade (P = 0.030), preMAI imbalance (P<0.001), postMAI imbalance (P<0.001), and ΔMAI (P<0.001) were associated with reduced OS. Poor PFS was significantly correlated with advanced TNM stage (P = 0.001), poor differentiation grade (P<0.001), preMAI imbalance (P<0.001), postMAI imbalance (P<0.001), and ΔMAI (P<0.001).

**Table 4:** Univariate COX Regression Analysis on the Impact of Clinicopathological Features on OS and PFS.

Variables	OS			PFS		
	HR	95%CI	P value	HR	95%CI	P value
Age (years)			0.166			0.475
<65	Reference			Reference		
≥65	1.324	0.890-1.970		1.136	0.801-1.613	
Gender			0.395			0.730
Female	Reference			Reference		
Male	1.214	0.777-1.895		1.075	0.715-1.615	
Smoking history			0.495			0.980
Never	Reference			Reference		
Current or former	1.142	0.780-1.671		1.004	0.710-1.420	
Alcohol consumption history			0.568			0.945
Never	Reference			Reference		
Current or former	1.115	0.768-1.619		1.012	0.720-1.423	
KPS			0.584			0.459
90-100	Reference			Reference		
70-90	1.125	0.739-1.713		1.156	0.788-1.695	
Tumor location			0.593			0.888
Upper/Middle	1.114	0.751-1.653		0.975	0.683-1.391	
Lower	Reference			Reference		
TNM stage			0.002			0.001
II/III	Reference			Reference		
IV	1.891	1.255-2.848		1.893	1.281-2.800	
Pathological T stage			0.853			0.732
T1-2	Reference			Reference		
T3-4	0.950	0.551-1.639		0.915	0.549-1.523	
Pathological N stage			0.244			0.221
N0-1	Reference			Reference		
N2-3	1.251	0.858-1.825		1.241	0.878-1.753	
Differentiation grade			0.027			<0.001
Well/Moderate	Reference			Reference		
Poor	1.709	1.062-2.750		2.367	1.494-3.751	
NLR			0.171			0.101
<8.83	Reference			Reference		
≥8.83	0.767	0.524-1.121		0.745	0.525-1.059	
SII			0.066			0.199
<853.39	Reference			Reference		
≥853.39	0.704	0.484-1.024		0.797	0.564-1.127	
PNI			0.161			0.528
<49.63	1.307	0.899-1.900		1.116	0.794-1.568	
≥49.63	Reference			Reference		
CEA			0.668			0.672
<5ng/ml	Reference			Reference		
≥5ng/ml	0.912	0.600-1.387		0.920	0.625-1.354	
CA199			0.588			0.357
<34U/ml	Reference			Reference		
≥34U/ml	0.887	0.574-1.370		0.833	0.557-1.246	

HR, Hazard ratio; CI, Confidence interval; OS, overall survival; PFS, progression-free survival; KPS, Karnofsky Performance Status; TNM stage, Tumor Node Metastasis; NLR, neutrophil-to-lymphocyte ratio; SII, systemic immune-inflammation index, PNI, prognostic nutritional index CEA, carcinoembryonic antigen; CA199, carbohydrate antigen19-9.

**Table 5:** Univariate COX Regression Analysis on the Impact of MAI on OS and PFS.

Variables	OS			PFS		
	HR	95%CI	P value	HR	95%CI	P value
preMAI			<0.001			<0.001
Balance	Reference			Reference		
Imbalance	2.772	1.784-4.309		2.588	1.758-3.809	
postMAI			0.005			0.064
Balance	Reference			Reference		
Imbalance	1.974	1.226-3.117		1.446	0.979-2.136	
ΔMAI			<0.001			<0.001
0 group	Reference			Reference		
1 group/2 group	4.825	3.245-7.175		3.622	2.530-5.185	

HR, Hazard ratio; CI, Confidence interval; OS, overall survival; PFS, progression-free survival; preMAI, Pre-therapy Muscle-Adipose Index; postMAI, Post-therapy Muscle-Adipose Index; ΔMAI, Change in Muscle-Adipose Index.

Based on the results of univariate analysis, TNM stage, tumor differentiation grade, preMAI, postMAI, and ΔMAI were incorporated as covariates into the multivariate cox regression analysis. PostMAI (HR = 2.617; 95%CI=1.159-5.910; P = 0.021) and ΔMAI (HR = 2.953; 95%CI=1.070-8.151; P = 0.037) independently predicted OS. Differentiation grade (HR = 1.643; 95%CI=1.017-2.655; P = 0.042) and ΔMAI (HR = 3.204; 95%CI=1.166-8.806; P = 0.024) were independently associated with PFS (**Table 6**).

**Table 6:** Multivariate Cox regression analysis of patient OS and PFS.

Variables	OS			PFS		
	HR	95%CI	P value	HR	95%CI	P value
TNM stage			0.071			0.035
II/III	Reference			Reference		
IV	1.471	0.968-2.237		1.541	1.030-2.304	
Differentiation grade			0.266			0.004
Well/Moderate	Reference			Reference		
Poor	1.330	0.805-2.197		2.018	1.254-3.246	
preMAI			0.131			0.296
Balance	Reference			Reference		
Imbalance	0.544	0.247-1.200		0.296	0.797-2.105	
postMAI			0.062			
Balance	Reference					
Imbalance	0.454	0.198-1.042				
ΔMAI			<0.001			<0.001
0 group	Reference			Reference		
1 group/2 group	9.260	3.733-22.971		2.713	1.747-4.212	

HR, Hazard ratio; CI, Confidence interval; TNM stage, Tumor Node Metastasis; OS, overall survival; PFS, progression-free survival; preMAI, Pre-therapy Muscle-Adipose Index; postMAI, Post-therapy Muscle-Adipose Index; ΔMAI, Change in Muscle-Adipose Index.

## Discussion

In this study, we evaluated the prognostic value of preMAI, postMAI and ΔMAI in inoperable ESCC patients undergoing chemoradiotherapy. Our analysis revealed that preMAI balance, postMAI balance, and ΔMAI 0 group were significantly associated with better clinical outcomes in inoperable ESCC patients. Furthermore, ΔMAI emerged as an independent prognostic factor for OS and PFS.

Previous studies have demonstrated that nutritional status significantly impacts treatment efficacy, quality of life, and prognosis in cancer patients (17, 18). To further elucidate the potential relationship between nutritional status and cancer outcomes, as well as to enable early identification and intervention for malnutrition, scientists have investigated various nutritional indicators and their prognostic implications in cancer (19). SII, NLR, PLR, and lymphocyte-to-monocyte ratio (LMR), as markers of systemic inflammation, have been associated with prognosis across different cancer types (20).Elevated SII levels are significantly correlated with an increased overall cancer risk (21). In colorectal cancer patients, high SII is linked to poorer disease outcomes, including worse OS (HR = 1.75; 95%CI=1.40-2.19) and PFS (HR = 1.25; 95%CI=1.18-1.33) (22, 23). A study by Tan et al. revealed that elevated NLR and PLR were associated with inferior OS and PFS in gastric cancer patients treated with immune checkpoint inhibitors (ICIs), whereas high LMR correlated with improved OS and PFS (24). Meghan et al. conducted a meta-analysis demonstrating that high NLR generally predicts poorer survival and a higher risk of disease progression (25). Xu et al. retrospective analysis of 771 gastric cancer patients undergoing radical resection identified SII and PNI as independent prognostic markers, suggesting their preoperative assessment could aid high-risk patient identification and treatment strategy optimization (26). The CONUT score-integrating inflammatory, nutritional, and immune parameters-has demonstrated superior prognostic value for colorectal cancer patients in recent years (27, 28).

However, the above indicators are derived from hematological tests, which mostly provide indirect and nonspecific assessments of nutritional status. They are prone to interference from confounding factors, have rapid dynamic changes, and exhibit poor reproducibility. In contrast, CT imaging allows for the direct quantification of skeletal muscle mass and adipose tissue, which can specifically reflect the composition of the body and long-term nutritional status, offering greater stability (29, 30). Accumulating evidence highlights body composition as a critical nutritional indicator closely linked to long-term outcomes in cancer patients (31, 32). SMI, a key indicator of skeletal muscle mass, has multidimensional clinical importance in the prognosis of patients with malignant tumors. Low SMI is indicative of sarcopenia, which has been significantly associated with reduced survival rates in patients with hepatocellular carcinoma (33), non-small cell lung cancer (34), colorectal cancer (35), and ovarian cancer (36). However, it is important to acknowledge that SMI and fat indices exhibit substantial variability across different geographic and ethnic populations, and currently there is a lack of universally accepted cutoff values (37, 38). This limits direct comparison between studies and highlights the need for population-specific standards.

Adiposity and its distribution are strongly linked to the prognosis of patients with malignant tumors, with mechanisms involving metabolic disorders, inflammatory responses, and immune dysfunction. Visceral adipose tissue (VAT) is an independent cancer risk factor beyond BMI, with a more significant impact in Asian populations (39). The Chinese visceral adiposity index (CVAI) can effectively predict cancer incidence, with the highest quintile of CVAI associated with a 2.81-and 2.85-fold increased risk of colorectal and breast cancer, respectively (40, 41). Abdominal fat is divided into visceral fat area (VFA) and subcutaneous fat area (SFA), which have distinct prognostic implications. In non-small-cell lung cancer, patients with high VFA combined with low SFA have the longest median survival (108 months) and the lowest systemic inflammatory indices (SII and AISI), suggesting VFA might improve prognosis via anti-inflammatory or metabolic protective mechanisms (42). Excess SFA, however, promotes systemic inflammation and counteracts the protective effects of VFA, likely mediated by hypoxia and fibrosis resulting from adipocyte hypertrophy (43). Mathias et al. reported that adipose accumulation correlated with increased risks of endometrial, renal, hepatic, and esophageal adenocarcinoma, particularly in females, while paradoxically reducing risks of ESCC, male lung cancer, and oral cavity cancers (44). In the present study, we focused specifically on subcutaneous adipose tissue due to its more straightforward quantification on CT imaging, established correlation with systemic metabolic and inflammatory states, and relevance in prior esophageal cancer literature. While visceral adipose tissue also holds prognostic significance, its measurement can be more variable and technically challenging. Our aim was to establish a reproducible and accessible metric for clinical use, though future studies incorporating both fat compartments may provide further insights. However, most prior studies have focused solely on the impact of single skeletal muscle or adipose indices on prognosis of cancers (45). In this study, we firstly present a novel metric termed MAI, which assesses skeletal muscle density or fat content to concurrently reflect muscle degradation and fat infiltration in ESCC. Our date demonstrated that preMAI imbalance was significantly associated with poor OS (P<0.001) and PFS (P<0.001). Additionally, postMAI imbalance implied poor OS (P<0.001) and PFS (P<0.001). Our multivariate analysis results found that postMAI was identified as an independent prognostic factor for OS (HR = 2.617; 95%CI=1.159-5.910; P = 0.021).

Dynamic monitoring of nutritional parameters during therapeutic interventions provides a more comprehensive nutritional assessment of cancer patients. Radiographically quantified ΔSMI dynamics (pre- to post-treatment skeletal muscle index changes) capture both muscle depletion progression and significant prognostic value in cancer patients (46, 47). Li et al. investigated perioperative ΔSMI in colorectal cancer patients and found that low SMI at baseline, 6, 9, and 12 months postoperatively predicted poorer OS and RFS (48). Additionally, combining SMI with other biomarkers may enhance prognostic or therapeutic predictions. Ji et al. suggested that pancreatic cancer patients with high ΔSMI and CA19-9 ≥37 U/mL may not be suitable for early local therapy and should instead continue chemotherapy (16). Despite these advances, the role of MAI in cancer prognosis remains underexplored, particularly beyond single preoperative timepoints in surgical cohorts. Notably, studies evaluating MAI changes before and after treatment are scarce. In our study, we analyzed the prognostic impact of ΔMAI in inoperable ESCC patients. Patients were stratified into three groups based on MAI balance status before and after treatment: 0 group(_pre_balance-_post_balance), 1 group (_pre_balance-_post_imbalance or _pre_imbalance-_post_balance) and 2 group (_pre_imbalance- _post_imbalance). Multivariate analysis confirmed that ΔMAI (0 group vs.1 group and 2 group) independently predicted with OS (HR = 2.953; 95%CI=1.070-8.151; P = 0.037) and PFS (HR = 3.024; 95%CI=1.166-8.806; P = 0.024).

While our findings suggest that CT-derived MAI holds promise as a radiological biomarker for assessing nutritional status and guiding personalized treatment, we acknowledge that these conclusions remain exploratory for several reasons. All measurements were conducted retrospectively, and the proposed cutoff values were derived from our cohort without external validation. The absence of standardized thresholds and the lack of validation in diverse populations limit the immediate clinical applicability and generalizability of our results. Therefore, we emphasize the need for prospective, multi-center studies to verify these cutoffs and establish robust, population-specific norms before clinical implementation.

This study has several limitations. First, its single-center retrospective design may introduce selection bias, potentially compromising objectivity and generalizability. Strict exclusion criteria further limited the cohort, possibly skewing patient characteristics. Second, our analysis exclusively included inoperable ESCC patients, necessitating further validation in surgical populations. Third, ΔMAI were assessed only at the first post-treatment follow-up, failing to account for longitudinal or time-dependent effects. Fourth, the absence of immunotherapy data may hinder a comprehensive evaluation of treatment efficacy. Finally, as noted above, the proposed MAI cutoffs require validation in independent and ethnically diverse cohorts to confirm their reproducibility and clinical relevance.

## Conclusion

This study demonstrated that CT-derived MAI hold independent prognostic value in inoperable ESCC patients treated with chemoradiotherapy. PostMAI imbalances significantly correlated with poorer survival outcomes, while ΔMAI magnitude better reflected long-term prognosis. Multivariate analysis identified ΔMAI as an independent predictors of survival, underscoring its potential role in risk stratification. Despite the inherent limitations of retrospective design, our findings support MAI as a radiologic biomarker for assessing nutritional status and guiding personalized therapy. Future studies should explore its interplay with inflammatory microenvironments.

## Data Availability

The raw data supporting the conclusions of this article will be made available by the authors, without undue reservation.
